# A Case Study of Cognitive Impairment Associated With Levetiracetam

**DOI:** 10.1192/bjo.2024.675

**Published:** 2024-08-01

**Authors:** Stefan McKenzie, Kalyan Seelam

**Affiliations:** South West Yorkshire Partnership NHS Foundation Trust, Barnsley, United Kingdom

## Abstract

**Aims:**

In patients with cognitive impairment, it is important to assess the possible impact of medications on cognition. Levetiracetam is an antiepileptic medication used in the management of epilepsy. Its effect on cognition is unclear.

**Methods:**

We present a case study of a 57-year-old female who developed cognitive impairment associated with levetiracetam.

She was referred to Memory Services from her GP due to cognitive impairment. Her past medical history included an optic nerve glioma which was surgically removed followed by radiotherapy, and meningiomas which were managed with stereotactic radiosurgery. She had no previous psychiatric history.

Following a first seizure, she was started on levetiracetam 250 mg BD. Over the following months, she developed worsening symptoms of poor memory, fatigue and lethargy, sleeping excessively, headaches, and subsequently, low mood and occasional suicidal thoughts. Levetiracetam dose was halved. When seen in Memory Services 3 months later, it was reported that there had been a gradual but partial improvement in her symptoms since the dose reduction. Addenbrooke's Cognitive Examination (ACE-III) score was 67/100. Short form mood scale was 3/15, below the threshold for depression. Blood tests were normal. MRI Head showed meningiomas and diffuse white matter hyperintensities, both unchanged from previous imaging.

The patient then started lamotrigine and stopped levetiracetam. On follow up (2 months after initial memory assessment), ACE-III score improved to 80/100 and it was reported that her symptoms had completely resolved.

**Results:**

In this case, there is evidence to support a causal link between levetiracetam and the patient's cognitive impairment – there was a temporal relationship, dose response relationship, and reversibility, which are all in the Bradford Hill criteria for causation. Other causes were considered and deemed less likely, including depression; the mood symptoms were not the predominant symptoms and developed after the other symptoms, and the patient scored below the threshold score for depression on short form mood scale.

Regarding the aetiology in this case, one hypothesis is that there may have been risk factors that made this patient more susceptible to cognitive side effects from the biological effects of levetiracetam, such as previous neurosurgery and radiosurgery. Another hypothesis is that the levetiracetam may have triggered an atypical depressive episode which manifested predominantly with memory symptoms and tiredness.

**Conclusion:**

This case study highlights the importance of reviewing medications when assessing cognitive impairment, and of obtaining a clear timeline of symptoms. There is a need for further research looking at the effect of levetiracetam on cognition.

